# MAG, myelin and overcoming growth inhibition in the CNS

**DOI:** 10.3389/fnmol.2015.00051

**Published:** 2015-09-07

**Authors:** Lisa McKerracher, Kenneth M. Rosen

**Affiliations:** ^1^BioAxone BioSciences Inc.Cambridge, MA, USA; ^2^Department of Neurology and Neurosurgery, McGill UniversityMontreal, QC, Canada

**Keywords:** myelin, myelin-associated glycoprotein, axon regeneration, myelin-derived inhibitors, neurotrauma, Rho, Nogo

## Abstract

While neurons in the central nervous system (CNS) have the capacity to regenerate their axons after injury, they fail to do so, in part because regeneration is limited by growth inhibitory proteins present in CNS myelin. Myelin-associated glycoprotein (MAG) was the first myelin-derived growth inhibitory protein identified, and its inhibitory activity was initially elucidated in 1994 independently by the Filbin lab and the McKerracher lab using cell-based and biochemical techniques, respectively. Since that time we have gained a wealth of knowledge concerning the numerous growth inhibitory proteins that are present in myelin, and we also have dissected many of the neuronal signaling pathways that act as stop signs for axon regeneration. Here we give an overview of the early research efforts that led to the identification of myelin-derived growth inhibitory proteins, and the importance of this family of proteins for understanding neurotrauma and CNS diseases. We further provide an update on how this knowledge has been translated towards current clinical studies in regenerative medicine.

## Introduction

Ramon y Cajal (1991), the Spanish neuroscientist who won the Nobel Prize in Medicine in 1906, first described in detail the frustrated growth response of axons injured in the spinal cord. Cajal observed in 1905 that cut axons first form growth cones similar to those seen in development of the nervous system, but then retract into round, static bulbs. Until the early 1980s it was assumed that there was a lack of an intrinsic ability of central nervous system (CNS) neurons to regenerate. Albert Aguayo first showed that CNS axons can regenerate their injured axons when provided with a peripheral nerve graft (Richardson et al., 1980; David and Aguayo, 1981). Later, Aguayo showed that regenerated fibers could form synapses and functional connections, even in adult animals (Keirstead et al., 1985). All of which raised the question: If CNS neurons were able to regenerate, what was it that was stopping them from doing so after an injury?

Advances in our understanding of the control of axonal regeneration have followed from the discovery of growth inhibitory molecules in the CNS, which can signal to block axonal extension. In its most basic form, CNS injury causes the release of fragmented, disrupted myelin, and this myelin debris inhibits neurite outgrowth. However, the discovery of this growth inhibitory activity was not confirmed until the individual myelin proteins responsible were identified (reviewed by Filbin, 2003). Following the description of these inhibitory proteins, the identification of their specific neuronal receptor molecules further increased support for the rather surprising finding that there existed an endogenous neuronal growth inhibition in the CNS.

## Myelin-Derived Inhibitors of Axon Growth

**Figure 1:**
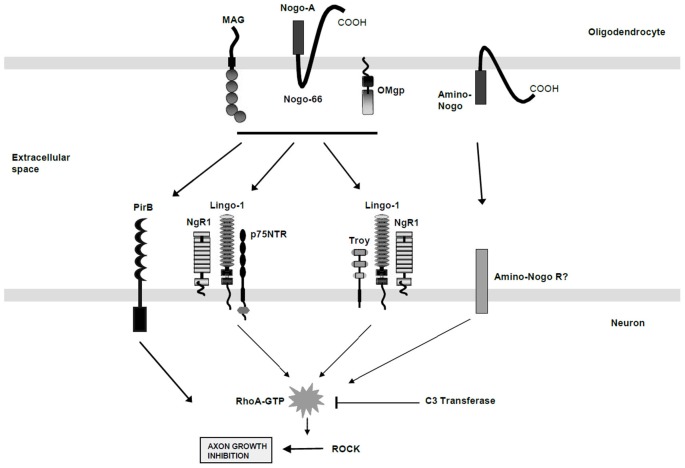
**A schematic diagram of the protein components involved in the myelin protein-derived inhibition of neurite growth.** The oligodendrocyte derived proteins released from the cell membrane following injury include MAG, Nogo-A (Nogo-66), Oligodendrocyte myelin glycoprotein (OMgp) and Amino-Nogo. The multicomponent cell surface receptor complexes that are localized to the neuronal cell membrane include the Nogo Receptor 1 (NgR1), Lingo-1, p75NTR, and TROY, all of which signal to change the activation of Rho (active Rho is indicated by RhoA-GTP). The PirB receptor also binds the myelin-derived inhibitory molecules but its absolute signaling pathway remains unclear. The C3 transferase enzyme, isolated from Clostridial species, is known to block these Rho activation pathways and to allow for neurite outgrowth in the face of myelin-derived protein inhibition. (Adapted by permission from Macmillan Publishers Ltd: J. Cereb. Blood Flow Metab.; Chaudhry and Filbin; 27(6):1096–107, Copyright 2007).

The inhibitors of CNS regeneration can be classified into three main categories: (1) Myelin-associated inhibitors; (2) Inhibitors associated with the glial scar that forms after injury; and (3) Inhibitors of the “guidance type”. We focus here on the myelin-derived inhibitors (see Figure 1). They are important because myelin is disrupted in both traumatic injury (Richardson et al., 1982) and neurological diseases including multiple sclerosis (Simons et al., 2014) and neurodegenerative disorders (Bartzokis, 2011).

## MAG and Inhibition of Neurite Outgrowth

The first myelin-associated CNS growth inhibitory protein to be identified was myelin-associated glycoprotein (MAG), and this activity was discovered independently by Marie Filbin and collaborators at Hunter College NY (Mukhopadhyay et al., 1994), and my group and collaborators at McGill University (McKerracher et al., 1994). The two studies used different approaches to discern that MAG has growth inhibitory activity, a surprising finding at the time because MAG is also expressed in peripheral nerve myelin, peripheral nerves regenerate naturally after injury, and it had previously been reported that MAG promoted neurite outgrowth (Johnson et al., 1989; Turnley and Bartlett, 1998).

My group at McGill was trying to understand why CNS nerves do not regenerate, and we had characterized changes in axonal transport and gene expression that were associated with CNS injury (Fournier and McKerracher, 1995, 1997). To understand how the environment of the CNS might inhibit regeneration, we set up a rapid neurite outgrowth assay using NG108 cells as a screening tool. We teamed up with two other McGill researchers, biochemist Peter Braun and neuroscientist Sam David, as we set out to purify the inhibitory proteins from myelin. We isolated myelin from calf brain and screened fractionated myelin proteins, seeking those proteins that would block neurite extension. We found several fractions that blocked neurite outgrowth, and the major peak of activity co-localized with the fractions where MAG eluted from the columns (McKerracher et al., 1994). Using purified recombinant MAG, we confirmed that MAG completely blocked neurite growth (McKerracher et al., 1994), and that growth cones retracted after making even a single filopodial contact (Shibata et al., 1998).

Simultaneously, and in complementary studies, Marie Filbin and collaborators Patrick Doherty and Frank Walsh at Guy’s Hospital in London discovered the growth inhibitory activity of MAG (Mukhopadhyay et al., 1994). Using a different approach, Filbin and colleagues were trying to understand how neurons interact with MAG by plating primary neurons on MAG substrates. To better simulate the presentation of MAG to primary neurons, they co-cultured neurons plated onto Chinese hamster ovary (CHO) cells expressing MAG on their surface. Surprisingly, they found that neurite outgrowth from cerebellar neurons was inhibited by MAG, an unexpected finding because it was initially believed that MAG promoted neurite outgrowth. When they tested dorsal root ganglion (DRG) neurons, they found that embryonic DRG neurons grew long neurites on the MAG-CHO cells, whereas MAG inhibited neurite outgrowth from neurons isolated from 7 day-old rats (Mukhopadhyay et al., 1994). These were the first studies to show a developmentally regulated switch in the neuronal response to myelin-derived growth inhibitory proteins, with MAG promoting the growth of embryonic neurons, while blocking axonal extension from postnatal neurons. Extending this line of studies, Filbin’s group went on to show that the developmental switch in growth responsiveness to myelin-MAG was regulated by the endogenous levels of cyclic AMP (cAMP; Cai et al., 2001). They identified a developmental decrease in neuronal cAMP levels and downstream PKA activity that was highly correlated with the change in neuronal responsiveness to MAG as it became an inhibitor of neurite outgrowth.

The finding that MAG was a growth inhibitory protein was both surprising and highly controversial at the time. Part of the reason for this was that Martin Schwab and collaborators were engaged in a search for a much larger, 250 kd growth inhibitory protein in the CNS (Caroni and Schwab, 1988b; Cloned 6 years after the identification of MAG, this was the myelin-derived inhibitor now known as Nogo). Immediately after the two papers reporting MAG as an inhibitor of regeneration (McKerracher et al., 1994; Mukhopadhyay et al., 1994), Melitta Schachner’s group, in collaboration with Martin Schwab, studied the myelin purified from MAG knockout mice, and determined that growth inhibitory activity remained (Bartsch et al., 1995). This paper showed that other inhibitors of neurite outgrowth were present in the myelin of MAG knockout mice, but it unleashed a fire-storm of controversy about the importance of MAG in growth inhibition, a controversy clearly explained in a classic paper published by Filbin (1996) entitled “The muddle with MAG”. Over the next decade Marie Filbin’s group carefully characterized the neuronal response to MAG and clarified the importance and role of MAG in growth inhibition in the CNS. My group went on to try to understand the neuronal signaling response to growth inhibitory proteins, focusing on Rho (Lehmann et al., 1999). Thus it is mainly the large body of work contributed by Filbin that has carefully characterized the neuronal response to MAG (for review, see Chaudhry and Filbin, 2007).

## MAG in Myelin and Released from Myelin

MAG is a minor constituent of myelin, and it is localized in mature, compact myelin only in the innermost membrane in contact with the axon (Trapp, 1990). It is present in both CNS and peripheral nervous system (PNS) myelin and is thought to be important for the long-term stabilization of myelinated axons (Fruttiger et al., 1995). Myelination is a relatively late event in neurodevelopment and therefore axonal growth cones would likely never come in contact with myelin, so it is not likely to play a role in stabilizing neuronal networks, as proposed for the other growth inhibitory proteins. Only after myelin is damaged and axons are attempting to regenerate would growth cones encounter MAG (Tang et al., 2001). In the PNS, where MAG may also be exposed after nerve injury, the high concentration of laminin is sufficient to override the growth inhibitory property of MAG (David et al., 1995). By contrast, the CNS does not contain much laminin or other favorable neuronal growth substrates.

Filbin made a major discovery about MAG and myelin-derived growth inhibition that, to date, has been underappreciated. She discovered that MAG becomes a much more potent component of CNS growth inhibitory activity after its release from damaged myelin in a diffusible form (Tang et al., 1997, 2001). Being a myelin biochemist, Filbin knew the historical literature on MAG, and the finding more than 10 years earlier that a soluble form of MAG exists, called dMAG (Sato et al., 1984). Filbin showed that the dMAG protein contains the entire extracellular domain of MAG, and it has a potent growth inhibitory activity. The Filbin group made use of MAG knockout mice to show that damaged white matter from normal but not MAG knockout mice inhibited neurite growth, and that immunodepletion of MAG from the soluble fraction removed the growth inhibition (Tang et al., 1997, 2001). These results show that MAG is the major inhibitor of axon growth that is released from damaged myelin and is present in the soluble fraction that can diffuse in the CNS and affect neurons that are not in direct contact with myelin debris. These findings have a major implication for other CNS diseases where myelin disruption occurs, such as multiple sclerosis, and could explain why, in the early phase of multiple sclerosis, neurons can degenerate without obvious evidence of frank myelin disruption, as soluble, released MAG fragments could be acting prior to the bulk collapse of myelin (Bjartmar and Trapp, 2001). Importantly, studies that leveraged knockout mice lines targeting myelin-associated inhibitor genes, either individually, or in combination, have left a certain amount of debate as to the precise role for each of these proteins (Ji et al., 2008; Cafferty et al., 2010; Lee et al., 2010). Much of the perceived difficulty relating to these mouse models likely can be ascribed to differences in the constructs targeting specific genes, the impact of the absence of these proteins throughout development, as well as the background of the mice on which they were generated. If nothing else, these studies have effectively shown that there is no simple answer to the role of myelin inhibitory proteins in the regeneration of the CNS for either localized regeneration or synaptic plasticity or the hope for growth of long axon tracts.

## Nogo

Despite the importance of MAG and other growth inhibitory proteins such as other myelin-derived proteins, chemorepulsive guidance molecules and proteoglycans present in the CNS, a great deal of recent research activity has focused on Nogo. Nogo’s extensive history in the regeneration literature is, in part, due to the assertion that it represents the majority of the growth inhibitory activity first detected by Martin Schwab. In this study he showed that neurons in culture could grow into peripheral nerves but not into explanted optic nerves (Schwab and Thoenen, 1985). These early findings led Schwab to hypothesize that a growth inhibitory activity exists in the CNS, and that a lack of axon regeneration was not simply due to a lack of appropriate growth factors.

The Schwab group created a monoclonal antibody, called IN-1, raised against an inhibitory fraction of myelin, and showed that it was able to attenuate the inhibitory properties of CNS myelin *in vitro* (Caroni and Schwab, 1988a). In adult rats, injection of the IN-1 antibody directly into the spinal cord promoted regeneration of axons in the corticospinal tract, and these axons grew past the lesion into the distal spinal cord (Schnell and Schwab, 1990). At a Neuroscience meeting in 1999, the Schwab group presented some peptide sequencing data from a high molecular weight protein, thought to be the elusive high molecular weight growth inhibitory protein. The cloning and sequencing of Nogo followed soon after (Chen et al., 2000; GrandPré et al., 2000; Prinjha et al., 2000). This Nogo protein was one of three isoforms (Nogo-A, Nogo-B and Nogo-C) produced from the Nogo gene by alternative splicing. Nogo-A is expressed in adult CNS neurons and oligodendrocytes but not in Schwann cells (GrandPré et al., 2000). Two inhibitory domains of Nogo have been identified: a 66 amino acid loop (Nogo-66) common to all three isoforms of Nogo and a unique amino-terminal region (amino-Nogo) specific to Nogo-A (Prinjha et al., 2000; Oertle et al., 2003). Although Nogo-A has been well characterized as a myelin-associated inhibitor for axonal regrowth in the injured CNS, the normal physiological function of Nogo-A in oligodendrocytes has yet to be fully elucidated. In Nogo knockout mice, delays in oligodendrocyte differentiation, myelin sheath formation and axonal caliber growth within the first postnatal month are observed, and the combined deletion of Nogo and MAG leads to transient hypomyelination (Pernet et al., 2008).

## OMgp

Oligodendrocyte myelin glycoprotein (OMgp) is a glycosylphosphatidylinositol (GPI)-anchored CNS myelin protein that is yet another myelin-derived protein that can inhibit neurite outgrowth (Kottis et al., 2002; Wang et al., 2002b). Intriguingly, OMgp is expressed in both neurons and oligodendrocytes in the CNS (Habib et al., 1998), and the expression of OMgp correlates with the onset of myelination (Mikol et al., 1990). OMgp plays a role in mediating the oligodendrocyte-oligodendrocyte and oligodendrocyte-axonal membrane interactions at the nodes of Ranvier (Mikol et al., 1990). Later evidence found a role for OMgp in oligodendroglial-like cells in preventing collateral sprouting and determining the spacing of the nodes of Ranvier (Huang et al., 2005).

## Neuronal Receptors for Growth Inhibitory Proteins

The identification of extracellular fragments of myelin-derived proteins as inhibitors of axonal regeneration in the CNS supported the notion of the existence of cell surface receptor molecules that would be involved in transmitting the growth inhibitory signal. The receptors for the various myelin-derived growth inhibitory proteins have taken significantly longer to identify, and new receptors/components are still being added to the list. One difficulty with identifying potential receptors is that it appears that MAG, Nogo and OMgp signal through receptor complexes on the neuronal membrane that may have a different array of constituents dependent upon the specific type of neuron being examined. Importantly, however, it appeared that MAG, Nogo and OMgp all signaled their inhibitory commands through a receptor complex containing, at a minimum, the Nogo-66 receptor NgR1 (Fournier et al., 2001; Domeniconi et al., 2002; Wang et al., 2002b). But given that NgR1 is a GPI-linked cell surface protein, additional partners are needed to transmit an intracellular signal inhibiting axonal outgrowth.

Continuing research into the nature and constituents of the neuronal receptor for myelin-derived growth inhibitors has now led to the identification of multiple proteins as potential participants. Work performed in the laboratory of Zhigang He identified the neurotrophin receptor molecule p75NTR as a component of a complex involving NgR1 (Wang et al., 2002a). Yet, other studies failed to identify an activation of downstream inhibitory Rho signaling if only NgR1 and p75NTR were expressed, which ultimately led to the identification of another component of this inhibitory signaling receptor complex, LINGO-1 (Mi et al., 2004). As the search for additional components continued, it was abundantly clear that multiple populations of CNS neurons expressed either very little or no detectable p75NTR. Research to address this question led to the identification of the TNF receptor family member TROY as yet an additional participant in an inhibitory signal transducing receptor complex (Park et al., 2005; Shao et al., 2005). Importantly, numerous studies have made it clear that all of these proteins are involved in signaling their inhibitory message to Rho. More recently, an additional transmembrane receptor protein has been identified as a high affinity binding site for myelin-derived inhibitory proteins, the paired immunoglobulin receptor B protein (PirB). Originally identified in the nervous system as an important modulator of visual cortical developmental plasticity (Syken et al., 2006), collaborative work between the Shatz and Tessier-Lavigne labs showed that all three myelin-derived inhibitory molecules bind to PirB with high affinity and it signals to inhibit neurite outgrowth (Atwal et al., 2008). The linkage between these inhibitory receptor complexes and CNS plasticity is one that continues to be explored, especially in the context of managing repair in the CNS.

## Translation to Clinical Studies

Advances in our understanding of the role of growth inhibitory protein in blocking axon regeneration and functional repair in the CNS have led to the development of viable drug candidates to treat neurotrauma. Clinical studies with MAG as drug target to promote nerve repair have been carried out by GlaxoSmithKline. They have studied the use of anti-MAG antibodies for potential efficacy in treating stroke. A humanized monoclonal antibody to MAG, GSK249320, blocks growth inhibition by MAG. This antibody was tested in 42 squirrel monkeys +24–72 h after stroke (Barbay et al., 2015). Trained on a pellet retrieval task, the monkeys had 30 mg/kg anti-MAG administered intravenously once weekly for 7 weeks. Treated animals showed a more rapid recovery of dexterity, with increased performance as early as 3 days after treatment. It is likely too early to be a result of axon regeneration, suggesting a neuroprotective effect (Barbay et al., 2015). Nogo receptor signaling to Rho plays an important role in neuroprotection (Dubreuil et al., 2003). Therefore blocking MAG inhibitory activity though binding the Nogo receptor likely accounts for the early recovery by reducing cell death. Further, mutant mice lacking NgR also recover from stroke better than controls (Lee et al., 2004).

Overall, MAG and other targets in the growth inhibitory signaling pathway show promise for translation to clinical study for treatment of various disorders, from multiple sclerosis and Alzheimer’s Disease to spinal cord injury (for review, see Hawryluk et al., 2008; Schwab, 2010; Schmandke et al., 2014).

## Summary

Today it is known that there is a plethora of growth inhibitory proteins expressed in the CNS, from the myelin-derived growth inhibitory proteins to chemorepulsive factors that are important in axonal guidance in development. The role of many of these proteins is still being elucidated, and it is now clear that growing axons respond to both “stop” and “go” signals, and that the preponderance of stop signals in the CNS plays a key role in preventing repair after traumatic injury. These findings have led to a new generation of approaches to overcoming growth inhibition in the CNS to promote regeneration and functional repair after injury. Marie Filbin’s pioneering work on MAG has both withstood the test of time and led the way for clinical advances to treat many different neurological disorders.

## Conflict of Interest Statement

Dr. Lisa McKerracher is the Founder and CEO of BioAxone BioSciences Inc. and Dr. Kenneth Rosen is an employee of BioAxone. BioAxone BioSciences is a company that is developing compounds to treat spinal cord injury.
